# Microbiological assessment of maize ogi cofermented with pigeon pea

**DOI:** 10.1002/fsn3.651

**Published:** 2018-05-09

**Authors:** Adebukunola Mobolaji Omemu, Uchechukwu Ifeoma Okafor, Adewale O. Obadina, Mobolaji O. Bankole, Samuel Ayofemi Olalekan Adeyeye

**Affiliations:** ^1^ Department of Hospitality and Tourism Federal University of Agriculture Abeokuta Nigeria; ^2^ Department of Microbiology Federal University of Agriculture Abeokuta Nigeria; ^3^ Department of Food Science & Technology Federal University of Agriculture Abeokuta Nigeria; ^4^ Department for Management of Science and Technology Development Ton Duc Thang University Ho Chi Minh City Vietnam; ^5^ Faculty of Environment and Labour Safety Ton Duc Thang University Ho Chi Minh City Vietnam

**Keywords:** cofermentation, maize, microorganisms, ogi, pigeon pea

## Abstract

Maize was cofermented with pigeon pea for *ogi* production and evaluated for microbiological qualities. White maize and pigeon pea were mixed at ratios of 90:10, 80:20, 70:30, 60:40, and 50:50, respectively, with 100:0 serving as the control. Mixtures were cofermented for 96 h at 27 ± 2°C, and microbiological and sensory qualities analyzed were carried out using analysis of variance. Values were significant at *p* ≤ .05. Results showed that there was a gradual decrease in the pH and increase in total titratable acidity (TTA), respectively, during fermentation in all the samples. At the end of fermentation, pH ranged from 3.47 to 4.27 and TTA ranged from 0.47% to 0.54%, respectively. Total heterotrophic count (THPC) ranged from 5.76 to 5.90 log cfu/g; lactic acid bacteria (LAB) from 6.15 to 5.98 log cfu/g; and yeasts from 5.51 to 5.79 log cfu/g. Microorganisms isolated were lactic acid bacteria (*Lactobacillus buchneri*,* L. casei, L. pentosus, Pediococcus pentosaceus*), yeasts (*Saccharomyces cerevisiae, Candida kefyr, C. krusei, C. tropicalis*), molds (*Aspergillus niger, A. flavus, Penicillium oxalicum, Mucor racemosus* and *Rhizopus stolonifer*) and other aerobic bacteria (*Klebsiella oxytoca, Enterobacter amnigenus, Staphylococcus xylosus, Bacillus subtilis, B. firmus, Corynebacterium kutscheri, C. striatum*, and *C. afermentans*). In conclusion, the total heterotrophic plate count (THPC) in fortified maize: pigeon pea products was very high. This could constitute health hazards to infants as weaning foods. However, the microbial loads could be reduced through heat treatment as ogi is usually boiled or treated with boiled water before consumption and it can therefore be concluded that the fortified maize: pigeon pea products could be used as weaning foods.

## 1 INTRODUCTION

Ogi is a popular fermented food in Nigeria and some parts of West Africa, made from maize (*Zea mays*), sorghum (*Sorghum vulgare*), or millet (*Eleusine coracana*) (Oyarekua, 2009). Unit operations involved in ogi preparation include soaking or steeping, wet milling, wet sieving, souring, and drying, if desired in the powdered form. Upon consumption, it is mixed with hot boiling water or cooked till it forms a thick gel. The color of maize ogi depends on the color of the cereal used. Yellow maize gives a cream‐colored ogi while white maize gives a white‐colored ogi.

Ogi is consumed by adults and children as breakfast meals, and it also serves as a weaning diet (Amusa et al., 2005; Ashaye, Fasoyiro, & Kehinde, 2000). After 5–6 months, breastfeeding is no longer sufficient to satisfy the nutritional requirements of the growing infant. Beginning from this period, the child needs solid foods to meet increasing nutritional needs (Onofiok & Nnanyelugo, 1998). This period is the weaning period, and in Nigeria, ogi (alternatively called pap or akamu) is introduced gradually to the child's diet to supplement nutrition. Fermented maize is very widely utilized as food in African countries, and in fact, cereals account for as much as 77% of total caloric consumption (Osungbaro, 2009). Maize is rich in carbohydrates and minerals, including potassium and magnesium. It, however, contains trace amounts of amino acids mainly lysine and tryptophan, contributing to the low content of protein, and trace amounts of vitamins, especially the B‐vitamins (USDA, 2012).

Lactic acid bacteria (*Lactobacillus plantarum* and *Streptococcus lactis*) and yeasts (*Saccharomyces cerevisiae, Rhodotorula* spp., *Candida mycoderma,* and *Debaromyces hansenii*) have been shown to be predominantly involved in fermentation of ogi, playing important roles as aroma development, microbial stability and flavor enhancement (Omemu et al., 2011; Aworh, 2008). Lactic acid fermentation also plays important roles in reducing antinutritional factors, increasing nutrient density and antimicrobial activities in the fermented product (Oyarekua, 2013). During ogi manufacturing, nutrients including protein and minerals are lost from the grains, thereby affecting its nutritional quality adversely (Ajanaku, Ajanaku, Edobor, & Nwinyi, 2012; Aminigo & Akingbala, 2004; Omemu, 2011).

The objective of this study is to evaluate the microbiological qualities of ogi developed from maize and pigeon pea.

## 2 MATERIALS AND METHODS

### 2.1 Sample collection

The pigeon pea (*Cajanus cajan*) and the maize grains (*Zea mays*) used in this study were purchased from Bariga market, Bariga, Lagos State, Nigeria.

### 2.2 Cleaning and weighing of maize/pigeon pea samples

The grains/peas were sorted to separate them from stones, dirt, etc. Six different proportions of maize: Pigeon peas were prepared as in Table 1.

**Table 1:** Materials used for the production of weaning food

Sample ratio	Maize (g)	Pigeon pea (g)
100:0	1000	0
90:10	900	100
80:20	800	200
70:30	700	300
60:40	600	400
50:50	500	500

### 2.3 Fermentation of maize–pigeon pea blends

**Figure 1:**
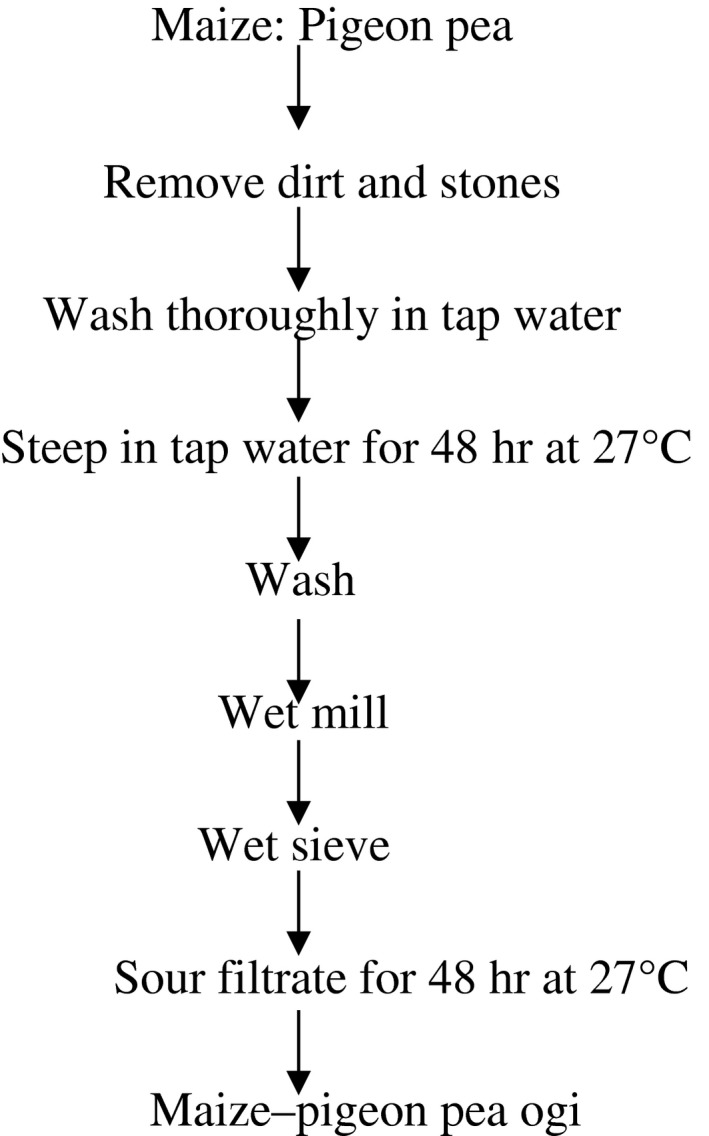
Unit operations for the production of Maize–pigeon pea ogi

The grains/peas were washed thoroughly and steeped in tap water in the ratio of 1:2 (w/v) in properly labeled plastic buckets with lids. Maize–pigeon pea ogi was prepared using a modified traditional fermentation method (Odunfa & Adeleye, 1985). The different maize–pigeon pea blends were steeped for 48 h, washed, wet milled, and then sieved using muslin cloth to separate the pomace from the filtrate. The filtrates were allowed to settle and sour for another 48 h (Figure 1). During the fermentation (steeping and souring) process, samples were taken at 24‐h interval for microbiological, chemical, nutritional, and antinutritional analysis.

### 2.4 Acidity changes during fermentation of the different maize–pigeon pea blends

The pH and total titratable acidity (TTA) of the different maize–pigeon pea blends were determined at 24‐h interval during fermentation.

### 2.5 pH

**T**he pH of the fermenting maize–pigeon pea blends was determined at 24‐h interval throughout the 96‐h fermentation period by the method of Omemu (2011).

### 2.6 Microbiological analysis

#### 2.6.1 Isolation of microorganisms

Samples (1 g) of fermenting maize–pigeon pea ogi were collected aseptically from the different buckets at 24‐h interval and homogenized in a mortar, previously cleaned with ethanol and passed over Bunsen flame. The homogenized samples were suspended in sterile 9 ml distilled water tubes and serially diluted (10‐fold dilution). Dilutions (0.1 ml) of 10^−3^–10^−5^ were inoculated on sterile disposable petri dishes by pour plate method. The plates were previously labeled appropriately based on the different media used; deMann Rogosa Sharpe (MRS) agar for lactic acid bacteria (LAB), plate count agar (PCA) for aerobic bacteria, HiCrome agar for *Bacillus* sp., MacConkey agar for enteric bacteria, yeast extract agar (YEA) for yeasts, and Sabouraud Dextrose Agar (SDA) for molds. The plates were then incubated appropriately to allow growth of organisms: MRS at 37°C in an anaerobic jar for 48 h, PCA at 37°C for 18–24 h, HiCrome and MacConkey agar at 37°C for 24 h, and YEA and SDA at 25°C for 3 days. Counts of bacteria, yeasts, and molds were made on the respective media. Microorganisms isolated at 24‐h interval during the fermentation (steeping and souring) process were randomly picked based on colonial morphology differences and subcultured onto freshly prepared plates by streaking. The isolates were purified by repeated subculturing and stored in agar slants at 4°C.

#### 2.6.2 Preservation of isolates

The purified isolates were kept on slants of their respective growth agar as stock cultures under refrigeration temperature. The isolates were subcultured and transferred unto fresh agar slants at 2‐month interval.

### 2.7 Characterization and identification of isolates

The identification of isolates was carried out in four phases; cultural characterization, morphological characterization, biochemical characterization, and the use of Analytical Profile Index (API) kits.

#### 2.7.1 Cultural characterization

Grouping of pure cultures of bacteria and yeasts randomly picked from inoculated plates was carried out on the basis of their colonial characteristics such as colony elevation, color, size, shape, surface, edge, and pigmentation. Macroscopic identification of molds was carried out by observing and recording the colony form, surface color, and pigmentation on plates.

#### 2.7.2 Morphological characterization

This was done by examining the isolates microscopically for cellular morphology. Day‐old cultures of the bacteria isolates were gram‐stained (section 3.6.2.1), and their color (purple or pink), shape (cocci or rods), and arrangement (singles, pairs, chains, or clusters) were observed and recorded. Day‐old cultures of the yeast and mold isolates were stained with lactophenol cotton blue and observed microscopically for cell shape, size, and sporulation. The method of vegetative reproduction (fission, filaments or budding) of the yeast isolates was also observed.

#### 2.7.3 Gram staining

Using a sterile loop, a light suspension of organism in sterile distilled water was prepared on a clean microscope slide. The film was air‐dried and heat‐fixed by passing the slide twice through a gas flame. The slide was then allowed to cool. The slide was placed on a staining rack, flooded with crystal violet solution, and left for 30 s before washing off with running tap water. The slide was again flooded with Lugol's iodine solution and left for 30 s before washing off with running tap water. To decolorize, acetone alcohol was run over the film and washed off immediately with running tap water. The film was flooded with safranin solution and left for 1 min before washing off with running tap water. The film on the slide was allowed to air‐dry. A drop of immersion oil was then placed on the film, and it was examined under the microscope using the ×100 oil immersion lens. Dark purple indicated Gram‐positive reaction and pink indicated Gram‐negative reaction. The shapes and arrangement of the cells were also recorded.

#### 2.7.4 Biochemical characterization

Conventional biochemical tests were carried out on the bacterial and yeast isolates for further identification such as catalase test, oxidase test, indole test, methyl red test, Voges–Proskauer test, citrate utilization, urease test, nitrate reduction, starch hydrolysis, casein hydrolysis, spore test, and sugar fermentation test.

##### Catalase test (slide method)

Catalase is an enzyme produced by microorganisms that live in oxygenated environments to neutralize toxic forms of oxygen (H_2_O_2_) for their protection. Anaerobes generally lack this enzyme, and yeasts are catalase positive. This test was carried out to distinguish Staphylococci and *Bacillus* spp. which are catalase positive and to identify the Enterobacteriaceae family. A drop of 3% hydrogen peroxide (H_2_O_2_) was placed on a clean glass microscopic slide. Using a sterile inoculating loop, a small amount of organism was picked from a well‐isolated 18‐ to 24‐h colony and gently rubbed into the H_2_O_2_. Positive reactions were evident by immediate bubble formation (Cheesbrough, 2006).

##### Oxidase test

This test was used to identify bacteria that produce cytochrome c oxidase, an enzyme of the bacterial electron transport chain. It was also used to identify *Candida* spp. which are yeasts and are oxidase positive. A piece of filter paper in a Petri dish was moistened with 2–3 drops of Kovac's oxidase reagent (1% tetramethyl‐*p*‐phenylenediamine). Using a wire loop, a colony of the test organism was transferred to the filter paper and rubbed on the moistened area. Purple coloration within 30 sec indicated the production of cytochrome c oxidase (Cheesbrough, 2006).

##### Indole test

This test was carried out to determine the ability of bacteria to break down tryptophan to indole by the enzyme tryptophanase. The test organism was inoculated in a bijou bottle containing 3 ml of sterile tryptone water and incubated at 35–37°C for 48 h. Indole was tested for by adding 0.5 ml (5 drops) of Kovac's reagent (isoamyl alcohol; para‐dimethyl aminobenzaldehyde; concentrated hydrochloric acid) and shaking gently. A red color in the surface layer within 10 min indicated a positive reaction while a yellow color indicated a negative reaction (Harrigan & McCance,^1976^).

##### Methyl red test

This test was carried out to identify enteric bacteria based on their pattern of glucose metabolism (mixed acid fermenters are positive to this test). The bacterium was inoculated into glucose phosphate broth, which contains glucose and a phosphate buffer and incubated at 37°C for 48 h. The pH of the medium was tested by the addition of five drops of methyl red reagent. The tube was gently rolled between the palms to disperse the methyl red reagent. Development of red color was taken as positive and yellow as negative (Somogyi, 1952).

##### Voges–Proskauer test

This test was carried out to detect acetoin in enteric broth culture with the aim of differentiating the enteric bacteria. The bacterium was inoculated into glucose phosphate broth and incubated for 48 h. Alpha‐naphthol solution (0.6 ml of 5%) in ethanol was added to the broth and shaken. The tube was allowed to stand for 15 min. Cherry red color was taken as positive while no color change indicated negative (Somogyi, 1952).

##### Citrate utilization test

This test was carried out to differentiate the enteric bacteria. Bacterial colonies from fresh (18‐ to 24‐h‐old) plates were picked up with wire loop, inoculated onto a slope of Simmons citrate agar and incubated overnight at 37°C. A change of medium from green to blue indicated a positive reaction, that is, the organism has the ability to utilize citrate as sole source of carbon and energy (Kiiyukic, 2003).

##### Urease test

This test was carried out to differentiate the enteric bacteria. Organisms positive to this test hydrolyze urea to produce ammonium ions with subsequent change in pH to alkaline (reaching 8.1) from an initial pH of 6.8. The test organism was inoculated heavily in a bijou bottle containing 3 ml sterile urea broth and incubated at 35°C for up to 7 days. Color change from yellow to rose pink was taken as positive (Harrigan & McCance, 1976).

##### Nitrate reduction test

This test was carried out to determine the ability of bacteria to reduce nitrate (NO_3_^−^) to nitrite (NO_2_^−^) using anaerobic respiration. Peptone water (5 ml) containing 0.1% potassium nitrate was dispensed in screw capped test tubes with Durham tubes and autoclaved at 121°C for 15 min. The test tubes were inoculated and incubated at 30°C for 72 h. Presence of gas in the Durham tubes indicated the production of nitrogen gas. Presence of nitrate was detected by the addition of 1 ml of a test solution containing equal volumes of 0.8% sulphanilic acid in 5N acetic acid and 0.5% alpha‐naphahthylamine in 5N acetic acid. Test tubes were shaken vigorously for about 2 min. Development of red coloration indicated a positive result. Small amounts of zinc dust were added to tubes still appearing negative to reduce any residual nitrate to nitrite (Payne, 1973).

##### Spore test

This test was carried out to identify the *Bacillus* spp. A film of the test organism was prepared on a clean microscope slide. The film was flooded with 10% aqueous malachite green solution, left to stand for 40–45 min, and washed under running tap water. It was again flooded with 0.5% aqueous safranin solution and left for 15 sec before rinsing under running tap water. The film was allowed to air‐dry. Bacterial bodies stained red and spores green (Somogyi, 1952).

##### H_2_S production

This test was carried out to determine the ability of the bacteria and yeast isolates to reduce sulfur‐containing compounds to sulfides during metabolism. The sulfide produces combines with iron compounds to produce ferrous sulfide (FeS), a black precipitate. Tubes of triple sugar iron (TSI) agar slopes were prepared, and using straight wire, the test organism was inoculated deep into butt of the medium and streaked up the slope. The tubes were incubated for 18–24 h at 37°C and examined for blackening of the medium (Somogyi, 1952).

##### Starch hydrolysis test

Starch agar (nutrient agar to which starch is added) plates were prepared and spot inoculated with test organisms. The plates were incubated at 35°C for 48 h. After incubation, the growth and surrounding areas was covered with Gram's iodine and the areas surrounding growth examined for clearing (Morello, Mizer, & Granato, 2003).

##### Casein hydrolysis test

Skim milk agar plates were inoculated with test bacteria and incubated at 35°C for 48 h. Zone of clearing around bacterial growth indicated the ability to secrete the proteolytic exoenzyme casesase that hydrolyze casein (Morello et al., 2003).

##### Sugar fermentation test

The tests were used to assess the sugar fermentation abilities of bacteria and yeasts isolated. Ten percent (10%) solutions of some test sugars (carbohydrates) such as glucose, fructose, lactose, galactose, mannitol, sucrose, raffinose, rhamnose, and melibiose were prepared and sterilized at 115°C for 10 min so as not to denature the sugars. To 90 ml sterile peptone water, 10 ml of the sterile 10% sugar solution was added with 1–2 ml 0.01% phenol red indicator. The sugar solution (5 ml) was transferred aseptically into sterile test tubes with inverted Durham tubes to check for gas production. The tubes were incubated overnight to check for sterility, and then, the tubes were inoculated with pure culture of the test organisms and incubated at 37°C for 4 days. D Yellow coloration indicates acid production while gas production was indicated by displacement of the medium in the Durham tube (Fawole & Oso, 2004).

### 2.8 Analytical profile index

Analytical profile index kits were used in confirming the identity of the isolates. The API kits used were API 20C AUX for yeast identification, API 50 CHL for lactic acid bacteria (LAB) identification, API CORYNE for Corynebacteria identification, API 20E for Enterobacteriaceae identification, API STAPH for Staphylococci identification, and API 50 CHB for *Bacillus* identification.

### 2.9 Analytical profile index (API 20C) for yeast identification

In the preparation of the inoculum, an ampoule of API suspension was opened and test colony was scooped with inoculating loop aseptically and transferred into the ampoule. This was mixed properly until the suspension became turbid. An ampoule of API C medium was opened, and approximately an aliquot of 100 l was transferred into the API C medium and gently homogenized. In the preparation of the API 20 C AUX strips, 5 ml of sterile distilled water was added into the honey‐combed wells of the incubation tray before the strip was placed in the incubation box. The cupules of the strip (0–29) were filled with the yeast suspension, obtained in the ampoule of API C medium, to the indicated inoculum level. The lid was placed onto the box, and the preparation was incubated at 29°C for 48–72 h, after which the strip was read and recorded in the result sheet. Identification was by the API identification system (apiweb).

### 2.10 Statistical analysis

Data obtained were subjected to analysis of variance (ANOVA) at α = 0.05 level of significance with the use of the Statistical Package for Social Sciences (SPSS) version 16.0. Significant means (*p* < .05) were separated using Duncan multiple range test. Graphs and charts were plotted with the use of Microsoft excel 2007 software.

## 3 RESULTS

### 3.1 Changes in pH of the fermenting maize–pigeon pea ogi blends

The changes in pH of the fermenting maize: pigeon pea blends during the 48‐h steeping and 48‐h souring process are presented in Table 2. The pH of all fermenting maize: pigeon pea blends ranged from 6.52 to 6.86 at 0‐h steeping period. The blend of 100:0 maize: pigeon pea had the lowest pH (6.52) while the 50:50 maize: pigeon pea blend had the highest pH (6.86). As fermentation progressed, the pH gradually decreased significantly (*p* ≤ .05) to 5.34–6.03 at the 48‐h steeping period. There was a slight increase in pH as souring (0‐h souring) began in all the maize: pigeon pea blends; however, the pH decreased as souring progressed (24‐h souring). At the 48‐h souring period which marked the end of the fermentation process, the 100:0 maize–pigeon pea blend had the lowest pH (3.74) while the 50:50 maize–pigeon pea blend had the highest pH (4.27).

**Table 2:** Changes in pH of the fermenting maize–pigeon pea ogi blends

Time (h)		Maize: pigeon pea proportion					
		100:0	90:10	80:20	70:30	60:40	50:50
Steeping phase	0	6.52 ± 0.01^f^	6.64 ± 0.02^e^	6.68 ± 0.02^d^	6.75 ± 0.02^c^	6.82 ± 0.02^b^	6.86 ± 0.02^a^
	24	6.17 ± 0.02^e^	6.21 ± 0.02^d^	6.33 ± 0.02^c^	6.36 ± 0.02^c^	6.47 ± 0.01^b^	6.54 ± 0.02^a^
	48	5.34 ± 0.01^f^	5.64 ± 0.02^e^	5.76 ± 0.02^d^	5.92 ± 0.02^c^	5.99 ± 0.01^b^	6.03 ± 0.02^a^
Souring phase	0	5.84 ± 0.02^f^	5.97 ± 0.01^e^	6.01 ± 0.02^d^	6.07 ± 0.02^c^	6.17 ± 0.02^b^	6.23 ± 0.02^a^
	24	3.90 ± 0.02^d^	4.20 ± 0.01^c^	4.22 ± 0.01^c^	4.29 ± 0.01^b^	4.30 ± 0.01^b^	4.38 ± 0.01^a^
	48	3.74 ± 0.03^e^	4.07 ± 0.02^d^	4.13 ± 0.02^c^	4.20 ± 0.01^b^	4.23 ± 0.02^b^	4.27 ± 0.02^a^

Values are mean ± standard deviation of triplicate determinations. Means on the same column with different sets of superscripts are statistically different (*p* ≤ .05).

### 3.2 Changes in total titratable acidity of the fermenting maize–pigeon pea ogi blends

**Figure 2:**
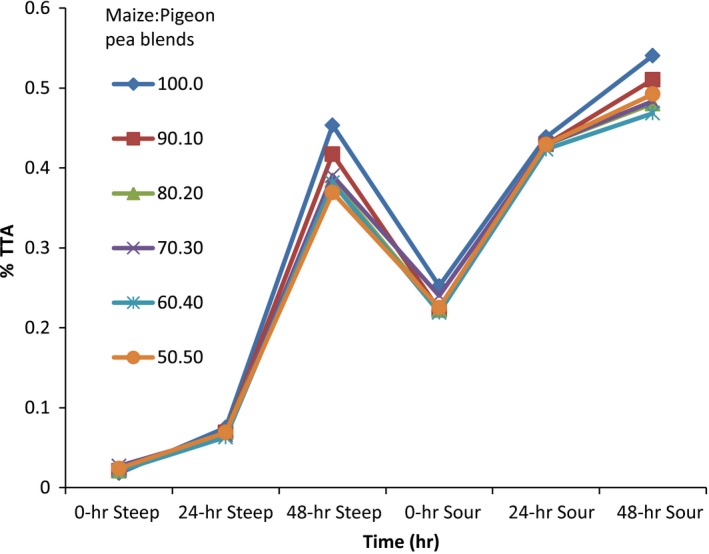
Total titratable acidity changes of the different fermenting maize–pigeon pea blends

The changes in total titratable acidity (TTA) of the fermenting maize: pigeon pea blends during the 48‐h steeping and 48‐h souring process are presented in Figure 2. At the 0‐h steeping period, the TTA of all fermenting mixtures ranged from 0.02% to 0.03%. The blend of 100:0 maize: pigeon pea had the highest TTA (0.03) while the 80:20, 70:30, 60:40, and 50:50 maize: pigeon pea blends had the lowest TTA (0.02).

As fermentation progressed, the TTA gradually increased significantly (*p* ≤ .05) in all the fermenting maize: pigeon pea blends, ranging from 0.37% to 0.45% at the 48‐h steeping period. There was a slight decrease in TTA at 0‐h souring in all the maize: pigeon pea blends; however, the TTA increased as souring progressed. At the 48‐h souring period which was the end of fermentation, the 100:0 maize: pigeon pea blend had the highest TTA (0.54), which was significantly (*p* ≤ .05) different from the others, while the 50:50 maize: pigeon pea blend had the highest TTA (0.47).

### 3.3 Microbial count

#### 3.3.1 Total heterotrophic plate count (THPC)

The total heterotrophic plate count (THPC) at the different phases of fermentation (steeping and souring) of all the maize–pigeon pea blends is shown in Table 3. At the 0‐h steeping period, the THPC ranged from 5.70 to 6.04 log cfu/g. As fermentation progressed, there was an observed significant (*p* ≤ .05) increase in the microbial count till the end of the steeping process (48‐h steeping). In contrast, at the 0‐h souring period, the total plate count was seen to significantly (*p* ≥ .05) decrease gradually till the end of souring. At the end of the souring phase, the THPC ranged from 6.03 log cfu/g in the 50:50 maize: pigeon pea blend to 6.26 log cfu/g in the 100:0 maize: pigeon pea blend.

**Table 3:** Total heterotrophic plate count (THPC) of the different fermenting maize–pigeon pea blends

Time (h)		Maize: pigeon pea proportion					
		100:0	90:10	80:20	70:30	60:40	50:50
Steeping phase	0	5.70 ± 0.74^e^	6.04 ± 0.88^e^	6.02 ± 0.78^d^	5.93 ± 0.76^d^	5.81 ± 0.76^d^	5.93 ± 0.88^d^
	24	6.02 ± 0.74^d^	6.18 ± 0.76^d^	6.20 ± 0.76^c^	6.22 ± 0.74^b^	6.23 ± 0.67^b^	6.42 ± 0.63^a^
	48	6.24 ± 0.84^c^	6.38 ± 0.83^a^	6.35 ± 0.76^a^	6.43 ± 0.83^a^	6.37 ± 0.80^a^	6.46 ± 0.83^a^
Souring phase	0	6.57 ± 1.34^a^	6.39 ± 0.83^a^	6.34 ± 0.80^a^	6.43 ± 0.85^a^	6.39 ± 0.81^a^	6.48 ± 0.84^a^
	24	6.40 ± 0.77^b^	6.29 ± 0.78^b^	6.26 ± 0.73^b^	6.21 ± 0.81^b^	6.23 ± 0.38^b^	6.26 ± 0.80^b^
	48	6.26 ± 0.64^c^	6.21 ± 0.65^c^	6.17 ± 0.68^c^	6.10 ± 0.69^c^	6.09 ± 0.32^c^	6.03 ± 0.30^c^

Values are mean ± standard deviation of triplicate determinations. Means on the same column with different sets of superscripts are statistically different (*p* ≤ .05).

#### 3.3.2 Total lactic acid bacteria count

The total LAB count at the different phases of fermentation (steeping and souring) of all the maize–pigeon pea blends is shown in Table 4. At the 0‐h steeping period, no lactic acid bacteria were isolated but at 24‐h steeping, and lactic acid bacteria were seen ranging from 5.47 log cfu/g in the 100:0 maize: pigeon pea blend to 4.97 log cfu/g in the 50:50 maize: pigeon pea blend. The lactic acid bacteria count significantly (*p* ≤ .05) increased in all the fermentation setups at 48‐h steeping. There was a nonsignificant (*p* ≥ .05) decrease in LAB count at the beginning of the souring process (0‐h souring) but the numbers significantly (*p* ≤ .05) increased appreciably as souring progressed. At the end of fermentation, lactic acid bacteria were seen ranging from 6.15 log cfu/g in the 100:0 maize: pigeon pea blend to 5.98 log cfu/g in the 50:50 maize: pigeon pea blend. A one‐way analysis of variance revealed that there was no significant effect (*p* ≥ .05) of fortification on the lactic acid bacteria count of the different fermentation setups.

**Table 4:** Lactic acid bacteria (LAB) Count of the different fermenting maize–pigeon pea blends

Time (h)		Maize: pigeon pea proportion					
		100:0	90:10	80:20	70:30	60:40	50:50
Steeping phase	0	–	–	–	–	–	–
	24	5.47 ± 0.66^d^	5.28 ± 0.58^d^	5.25 ± 0.54^d^	5.19 ± 0.63^d^	5.04 ± 0.35^d^	4.97 ± 0.35^d^
	48	5.65 ± 0.75^c^	5.51 ± 0.72^c^	5.37 ± 0.61^c^	5.26 ± 0.71^c^	5.15 ± 0.74^c^	5.03 ± 0.41^c^
Souring phase	0	5.61 ± 0.58^c^	5.49 ± 0.57^c^	5.32 ± 0.36^c^	5.21 ± 0.38^c^	5.10 ± 0.32^c^	4.97 ± 0.42^c^
	24	5.89 ± 0.84^b^	5.81 ± 0.82^b^	5.71 ± 0.75^b^	5.69 ± 0.76^b^	5.64 ± 0.71^b^	5.58 ± 0.69^b^
	48	6.15 ± 0.79^a^	6.11 ± 0.78^a^	6.09 ± 0.78^a^	6.04 ± 0.74^a^	6.02 ± 0.73^a^	5.98 ± 0.70^a^

Values are mean ± standard deviation of triplicate determinations. Means on the same column with different sets of superscripts are statistically different (*p* ≤ .05).

#### 3.3.3 Total yeast count

The total yeast count at the different phases of fermentation (steeping and souring) of all the maize–pigeon pea blends is presented in Table 5. At the 0‐h steeping period, no yeast was isolated but at 24‐h steeping, and total yeast count ranged from 4.95 to 5.07 log cfu/g. The yeasts increased significantly (*p* ≤ .05) in number in all the fermentation setups from this period till the end of the souring phase. At the 48‐h souring period which marked the end of the fermentation process, yeasts were seen ranging from 5.53 log cfu/g in the 100:0 maize: pigeon pea blend to 5.79 log cfu/g in the 50:50 maize: pigeon pea blend.

**Table 5:** Total Yeast count of the different fermenting maize–pigeon pea blends

Time (h)		Maize: pigeon pea proportion					
		100:0	90:10	80:20	70:30	60:40	50:50
Steeping phase	0	–	–	–	–	–	–
	24	4.98 ± 0.60^d^	4.97 ± 0.66^d^	4.98 ± 0.77^c^	4.98 ± 0.45^d^	4.95 ± 0.63^d^	5.07 ± 0.60^c^
	48	5.16 ± 0.78^c^	5.05 ± 0.71^c^	5.03 ± 0.73^c^	5.15 ± 0.82^c^	5.19 ± 0.77^c^	5.09 ± 0.68^c^
Souring phase	0	5.43 ± 0.77^b^	5.38 ± 0.62^b^	5.37 ± 0.67^b^	5.56 ± 0.74^b^	5.53 ± 0.75^b^	5.47 ± 0.64^b^
	24	5.51 ± 0.73^a^	5.52 ± 0.75^a^	5.57 ± 0.81^a^	5.58 ± 0.73^b^	5.56 ± 0.76^b^	5.51 ± 0.74^b^
	48	5.53 ± 0.77^a^	5.55 ± 0.73^a^	5.71 ± 0.80^a^	5.78 ± 0.77^a^	5.75 ± 0.57^a^	5.79 ± 0.64^a^

Values are mean ± standard deviation of triplicate determinations. Means on the same column with different sets of superscripts are statistically different (*p* ≤ .05).

#### 3.3.4 Mold count

**Figure 3:**
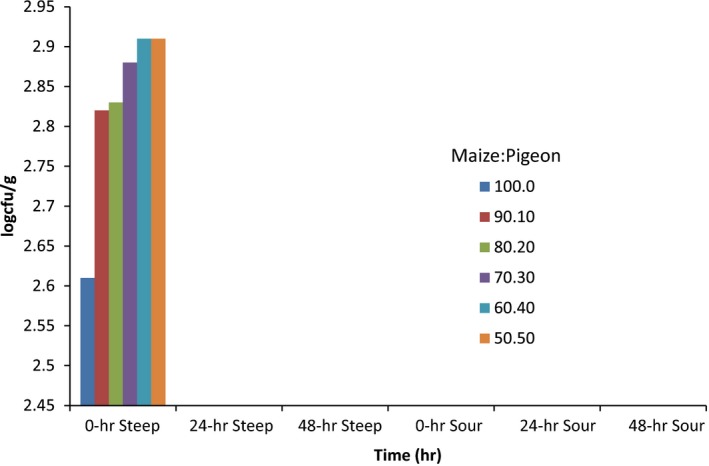
Mold count of the different fermenting maize–pigeon pea blends

The mold count at the different phases of fermentation (steeping and souring) of all the maize–pigeon pea blends is presented in Figure 3. At the 0‐h steeping period, molds encountered ranged from 2.81 log cfu/g in the 100:0 maize: pigeon pea blend to 2.91 log cfu/g in the 60:40 and 50:50 maize: pigeon pea blends. As fermentation progressed till the end of the fermentation process, there was a complete disappearance of molds.

#### 3.3.5 Coliform count

**Figure 4:**
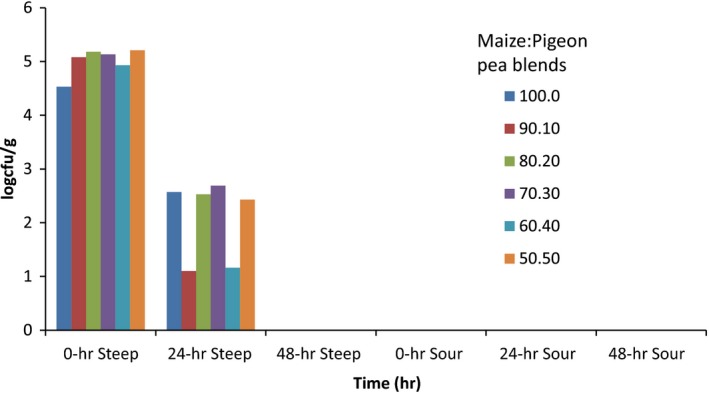
Coliform count of the different fermenting maize–pigeon pea blends

The coliform count at the different phases of fermentation (steeping and souring) of all the maize–pigeon pea blends is shown in Figure 4. At the beginning of fermentation, coliform count ranged from 4.53 log cfu/g in the 100:0 maize: pigeon pea blend to 5.21 log cfu/g in the 50:50 maize: pigeon pea blend. At 24‐h steeping, the count reduced significantly (*p* ≤ .05) to 1.10–2.69 log cfu/g. At 48‐h steeping and throughout the period of souring, there was a complete disappearance of coliform bacteria in all the fermentation setups.

#### 3.3.6 *Bacillus* count

The *Bacillus* count at the different phases of fermentation (steeping and souring) of all the maize–pigeon pea blends is shown in Table 6. At the 0‐h steeping period, *Bacillus* count ranged from 4.84 log cfu/g in the 100:0 maize: pigeon pea blend to 5.15 log cfu/g in the 50:50 maize: pigeon pea blend. As fermentation progressed, there was an observed significant (*p* ≤ .05) decrease in the count in all the fermenting mixtures. At the 48‐h souring period which marked the end of the fermentation process, no *Bacillus* was encountered in all the fermentation setups.

**Table 6:** *Bacillus* Count of the different fermenting maize–pigeon pea blends

Time (h)		Maize: pigeon pea proportion					
		100:0	90:10	80:20	70:30	60:40	50:50
Steeping phase	0	4.84 ± 0.80^a^	5.04 ± 0.76^a^	5.09 ± 0.80^a^	5.09 ± 0.82^a^	5.04 ± 0.73^a^	5.15 ± 0.77^a^
	24	2.67 ± 2.33^b^	4.69 ± 0.65^b^	4.59 ± 0.47^b^	4.57 ± 0.44^b^	4.58 ± 0.55^b^	4.70 ± 0.48^b^
	48	1.16 ± 2.01^c^	2.53 ± 2.20^c^	2.49 ± 2.17^c^	2.59 ± 2.28^c^	1.10 ± 1.91^c^	2.63 ± 2.31^c^
Souring phase	0	1.10 ± 1.91^c^	2.43 ± 2.14^d^	1.00 ± 1.73^d^	2.43 ± 2.14^d^	1.00 ± 1.73^d^	2.43 ± 2.14^d^
	24	1.00 ± 1.73^d^	1.00 ± 1.73^e^	1.00 ± 1.73^d^	1.00 ± 1.73^e^	1.00 ± 1.73^d^	1.00 ± 1.73^e^
	48	‐	‐	‐	‐	‐	‐

Values are mean ± standard deviation of triplicate determinations. Means on the same column with different sets of superscripts are statistically different (*p* ≤ .05).

### 3.4 Cultural, morphological, and biochemical characteristics of microorganisms isolated from the different maize: pigeon pea mixtures

The cultural, morphological, and biochemical characteristics of bacteria, yeasts, and molds isolated from the fermenting maize: pigeon pea mixtures using conventional method are shown in Tables 7, 8, and 9, respectively. A total of 12 bacteria, 16 yeasts, and 5 molds were isolated from the different fermenting maize: pigeon pea mixtures. The bacteria isolated include *Lactobacillus buchneri, L. pentosus, L. paracasei, Pediococcus pentosaceus, Bacillus subtilis, B. firmus, Klebsiella oxytoca, Enterobacter amnigenus, Staphylococcus xylosus, Corynebacterium kutscheri, C. striatum*, and *C. afermentans*, yeasts isolated include *Candida tropicalis, C. krusei, C. kefyr, and Saccharomyces cerevisiae* and molds isolated include *Aspergillus niger, A. flavus, Penicillium oxalicum, Mucor racemosus,* and *Rhizopus stolonifer*. The conventional method of biochemical characterization of microorganisms was used to determine the probable identity of the isolates.

**Table 7:** Cultural, Morphological and Biochemical characterization of bacterial isolates using conventional method

S/N	Code	Cultural characteristics	Cellular morphology	Gran reaction	Catalase	Oxidase	Indole	Motility	Methyl red	Voges–Proskaeur	Growth at 15°C	Growth at 45°C	NH_3_ from arginine	H_2_S production	Citrate utilization	Urease activity	Starch hydrolysis	Casein hydrolysis	NO_3_ reduction	Spore test	Glucose	Galactose	Sucrose	Maltose	Mannitol	Lactose	Xylose	Trehalose	Rhamnose	Raffinose	Melibiose	Fructose	Arabinose	Ribose	Probable Identity
**1**	L1	Cream; circular; raised; entire edge	Cocci in clusters	−	−	−	−	+	−	+	−	+	−	N	N	N	N	N	−	−	−	−	+	−	−	+	+	−−	‐	−	−	−	−	−	*Pediococcus pentosaceus*
**2**	L2	White; circular; raised; entire edge	Rods in pairs and clusters	−	−	−	−	+	−	+	−	+	−	N	N	N	N	N	−	−	+	+	+	−	+	−	+	−	−	−	−	+	−	−	*Lactobacillus buchnerii*
**3**	L3	Cream; circular; raised; entire edge	Rods in pairs and clusters	−	−	−	−	+	−	+	−	−	−	N	N	N	N	N	−	−	+	+	+	+	+	+	+	+	−	−	+	+	−	−	*Lactobacillus casei*
**4**	L4	Cream; circular; raised; entire edge	Rods in singles and clusters	−	−	−	−	+	−	+	−	−	−	N	N	N	N	N	−	−	+	+	+	+	+	+	−	−	+	+	+	+	+	−	*Lactobacillus pentosus*
**5**	H1	Pink; round; smooth shiny surface; entire edge; convex	Rods in clusters; spores present; flagellated	+	+	−	+	+	+	N	N	N	N	+	−	+	+	+	+	+	+	+	−	+	+	−	−	+	−	+	+	+	+	+	*Bacillus subtilis*
**6**	H2	Orange; round; smooth shiny surface; entire edge; flat	Rods in chains and clusters; spores present	+	+	−	+	+	−	N	N	N	N	−	−	+	+	−	+	+	−	+	+	+	−	−	−	−	−	+	−	−	+	+	*Bacillus formis*
**7**	P1	Yellowish orange; slimy; raised; smooth; serrated edge	Cocci in pairs or tetrads	+	+	−	−	−	+	−	+	−	−	−	+	+	−	−	−	−	+	+	+	−	−	+	+	+	−	−	+	−	+	+	*Staphylococcus xylosus*
**8**	P2	White; opaque; round; smooth; raised; entire	Straight rods in singles and pairs	+	+	−	−	−	−	−	−	+	−	−	+	+	−	+	−	−	−	+	+	−	−	−	+	−	+	−	−	−	+	+	*Corynebacterium kutscheri*
**9**	P3	White; opaque; round; smooth; raised; entire	Straight rods in singles and pairs	+	+	−	−	+	−	−	−	+	−	−	−	+	−	−	−	−	−	−	+	−	+	−	−	+	−	−	+	−	−	+	*Corynebacterium striatum*
**10**	P4	White; opaque; round; smooth; raised; entire	Straight rods in singles and pairs	+	+	−	−	−	−	−	−	+	−	−	+	−	−	+	−	−	−	+	+	−	+	−	−	+	−	−	+	−	−	+	*Corynebacterium afermentans*
**11**	P5	White and glistening; dome shaped; smooth; round; entire	Rods in singles, pairs and short chains	−	+	+	+	−	−	+	−	−	−	−	+	+	−	+	−	−	+	+	+	+	+	+	−	+	+	+	+	+	+	+	*Klebsiella oxytoca*
**12**	P6	Pink; smooth; flat; irregular	Rods in singles, pairs and clusters	−	+	−	−	+	−	+	−	−	−	−	+	+	−	+	−	−	−	+	+	+	+	+	−	+	+	−	−		−	+	*Enterobacter amnigenus*

**Table 8:** Cultural, Morphological, and Biochemical characterization of yeast isolates using conventional method

S/N	Code	Cultural characteristics	Cellular morphology	Catalase	Ascospore	Urease activity	NO_3_ reduction	Glucose	Galactose	Sucrose	Maltose	Mannitol	Lactose	Xylose	Trehalose	Rhamnose	Raffinose	Melibiose	Fructose	Arabinose	Ribose	Probable Identity
**1**	Y1	Cream, flat, dull, wrinkled surface, uneven	Cylindrical cells, true, septate hyphae	+	−		−	+	+	+	+	+	−	+	+	+	−	−	−	+	+	*Candida tropicalis*
**2**	Y2	Cream, flat, dull, wrinkled surface, uneven	Cylindrical cells, true, septate hyphae	+	−		−	+	+	+	+	+	−	+	+	+	−	−	−	+	+	*Candida tropicalis*
**3**	Y3	Cream, raised, glossy, entire edge	Oval to elongated, budding cells, no pseudomycelium	+	+		−	+	+	+	−	−	−	−	+	−	+	+	+	−	+	*Saccharomyces cerevisiae*
**4**	Y4	Cream, raised, glossy, entire edge	Oval to elongated, budding cells, no pseudomycelium	+	+		−	+	+	+	−	−	−	−	+	−	+	+	+	−	+	*Saccharomyces cerevisiae*
**5**	Y5	Cream, flat, smooth, glossy, entire edge	Short‐ovoid to long‐ovoid, budding blastoconidia, branched pseudohyphae	+	−		−	+	+	+	−	−	+	−	−	−	+	−	−	−	+	*Candida kefyr*
**6**	Y6	Cream, flat, dull, wrinkled surface, uneven	Cylindrical cells, true, septate hyphae	+	−		−	+	+	+	+	+	−	+	+	+	−	−	−	+	+	*Candida tropicalis*
**7**	Y7	Cream, flat, smooth, glossy, entire edge	Short‐ovoid to long‐ovoid, budding blastoconidia, no pseudohyphae	+	−		−	+	+	+	−	−	+	−	−	−	+	−	−	−	+	*Candida kefyr*
**8**	Y8	Cream, raised, glossy, entire edge	Oval to elongated, budding cells, no pseudomycelium	+	+		−	+	+	+	−	−	−	−	+	−	+	+	+	−	+	*Saccharomyces cerevisiae*
**9**	Y9	Cream, flat, smooth, glossy, entire edge	Short‐ovoid to long‐ovoid, budding blastoconidia, branched pseudohyphae	+	−		−	+	+	+	−	−	+	−	−	−	+	−	−	−	+	*Candida kefyr*
**10**	Y10	Cream, flat, dull, wrinkled surface, uneven	Cylindrical cells, true, septate hyphae	+	−		−	+	+	+	+	+	−	+	+	+	−	−	−	+	+	*Candida tropicalis*
**11**	Y11	Cream, flat, smooth, uneven edge	Small, elongated to ovoid budding blastoconidia, branched pseudohyphae	+	−		−	+	−	−	−	−	−	−	−	−	−	−	+	−	−	*Candida krusei*
**12**	Y12	Cream, flat, smooth, uneven edge	Small, elongated to ovoid budding blastoconidia, branched pseudohyphae	+	−		−	+	−	−	−	−	−	−	−	−	−	−	+	−	−	*Candida krusei*
**13**	Y13	White, flat, smooth, glossy, entire edge	Short‐ovoid to long‐ovoid, budding blastoconidia, no pseudohyphae	+	−		−	+	+	+	−	−	+	−	−	−	+	−	−	−	+	*Candida kefyr*
**14**	Y14	Cream, raised, glossy, entire edge	Oval to elongated, budding cells, no pseudomycelium	+	+		−	+	+	+	−	−	−	−	+	−	+	+	+	−	+	*Saccharomyces cerevisiae*
**15**	Y15	Cream, raised, glossy, entire edge	Oval to elongated, budding cells, no pseudomycelium	+	+		−	+	+	+	−	−	−	−	+	−	+	+	+	−	+	*Saccharomyces cerevisiae*
**16**	Y16	White, flat, smooth, glossy, entire edge	Short‐ovoid to long‐ovoid, budding blastoconidia, branched pseudohyphae	+	−		−	+	+	+	−	−	+	−	−	−	+	−	−	−	+	*Candida kefyr*

**Table 9:** Cultural and biochemical characterization of mold isolates using conventional method

S/N	Code	Colonial features	Conidiophores/sporangiophores	Type and color of spores	Types of hyphae	Special features	Identification
1	M1	Black with pale‐yellow reverse	Conidiophores arising from the substratum are long and smooth	Conidia are brown to black, very rough and globose,	Septate	Foot‐ cells	*Aspergillus niger*
2	M2	Dusty yellow at first, quickly becoming greenish yellow with fluffy and velvety texture; creamy reverse	Conidiophores are long and coarsely roughened	Green, globose conidia	Septate and branched	Foot‐ cells	*Aspergillus flavus*
3	M3	Dark‐green with margin shading through pale blue‐green to white; velvety; colorless to pale‐yellow reverse	Conidiophores are long and smooth	Smooth elliptical conidia with chains massed into columns	Septate	Foot‐ cells	*Penicillium oxalicum*
4	M4	White to grayish dense cottony masses of mycelium with white to pale reverse	Brown unbranched erect smooth‐walled sporangiophores	Black spores variously shaped, ellipsoidal, angular, striate in long axis	Branched nonseptate; broad	Brown rhizoids; spherical or elongated columella	*Rhizopus stolonifer*
5	M5	Dirty white, gray, or brownish gray; fluffy with white reverse	Short and erect sporangiophores; branched, hyaline	Colorless sporangiospores mostly ellipsoidal; zygospores arise from the mycelium	Broad nonseptate hyphae	Columella in varying shapes; no rhizoids	*Mucor racemosus*

## 4 DISCUSSION

There was a steady decrease in the pH during fermentation in all the ogi samples. This might be as a result of production of lactic acid by fermentative organisms (mainly lactic acid bacteria) responsible for the fermentation of ogi (Oluwafemi & Adetunji, 2011). A significant increase in the total titratable acidity (TTA) during fermentation in all the ogi samples was recorded in this study, and this could be as a result of production of lactic acid and other organic acids by organisms responsible for the fermentation, as is the case with the decrease in pH. This observation of decrease in pH and increase in total titratable acidity (TTA) agrees with most studies on ogi fermentation including those of Wakil and Kazeem (2012), Okwute and Olafiaji (2013), and Modu et al. (2013). The early rise in titratable acidity and reduced pH is important to avoid proliferation of undesirable organisms resulting in poor fermentation (Mbata, Ikenebomeh, & Alaneme, 2009).

This study revealed high total heterotrophic plate count (THPC) which may be attributable to availability of some easily metabolizable nutritional components of grains and legumes, which may be essential for the growth of microbes (Adebayo, Ogunsina, & Gbadamosi, 2013). A high total bacterial count has also been observed (Nsofor, Ume, & Uzor, 2014; Nwogwugwu, Ogbulie, Chinakwe, Nwachukwu, & Onyemekara, 2012) and reported to likely indicate a potential hazard to consumers. These counts are, however, lower than 1.0 × 10^8^ cfu/ml staphylococcal count normally considered as potentially hazardous (Nsofor et al., 2014). The THPC ranged from 6.03 log cfu/g in the 50:50 maize: pigeon pea blend to 6.26 log cfu/g in the 100:0 maize: pigeon pea blend. These values were too high for foods for infants that could be immune‐compromised people.

The increase in the lactic acid bacteria count with fermentation as observed in all the fermentation setups could be due to an increase in the acidity and the anaerobic condition of the fermenting medium, favouring the growth of only facultative anaerobes, and/or aciduric organisms. It may also have been as a result of the inhibitory effect of antimicrobial products from the lactic acid bacteria on the growth of unwanted harmful or spoilage organisms (Adebayo et al., 2013). Toxic substances such as hydrogen peroxide, diacetyl, carbon dioxide (CO_2_), organic acid, and bacteriocins have been shown to be released by lactic acid bacteria into the fermenting medium during food fermentation (Mataragas, Melaxopoulous, & Drosinos, 2002; Nsofor et al., 2014). These substances are toxic to pathogenic organisms that may be present in the fermenting substrates.

The lactic acid bacteria isolated in this study include mostly the *Lactobacillus* species (*Lactobacillus buchnerii, Lactobacillus casei, and Lactobacillus pentosus*). The higher prevalence of rod‐shaped lactic acid bacteria in this study corroborated the study of Nwokoro & Chukwu, 2012 who reported that the genus *Lactobacillaceace* commonly predominates during food fermentation. This is because they are the most aciduric of all lactic acid bacteria (Nsofor et al., 2014; Nwokoro & Chukwu, 2012).

Yeasts isolated in this study include *Saccharomyces cerevisiae, Candida kefyr,* and *Candida tropicalis*. Previous workers have found several yeasts species in spontaneous lactic fermenting cereals including species of *Saccharomyces* and *Candida* (Jespersen, Halm, Kpodo, & Jakobsen, 1994). The appearance and increase in the yeast count after 24 h of fermentation is attributed to the decrease in the pH that creates conditions ideal for yeast growth. This is similar with the finding reported for other fermented beverages (Abegaz, Beyene, Langsrud, & Narvhus, 2002). Roles of yeasts have also been reported to include improving the organoleptic qualities by producing different flavors and aroma in different foods (Inyang & Idoko, 2006).

The molds (*Aspergillus niger, Aspergillus flavus, Rhizopus stolonifer, Mucor racemosus,* and *Penicillium oxalicum*) isolated in the study are commonly present as contaminants in cereals and legumes and do not appear to play any significant important role in the fermentation (Mbata et al., 2009). The sources of these microorganisms could be human skin, cooking utensils, processing equipment, the environment and water (Ogbulie, 1991), or from the seeds. Jespersen et al. (1994) similarly reported the presence of molds such as *Penicillium* and *Aspergillus* in maize fermentation during kenkey production with drastic reduction in their numbers within 24 h of dough fermentation.

The subsequent disappearance of molds after 24 h observed in this study as well as previous studies (Mbata et al., 2009; Nsofor et al., 2014; Nwokoro & Chukwu, 2012) was probably due to the low oxygen tension in the fermenting matrix. It could also be due to the presence of organic acids especially lactic acid as it could be seen that the growth of lactic bacteria and yeasts increased gradually throughout fermentation while the numbers of molds decreased. Edema and Sanni (2008) also reported that the growth of lactic acid bacteria and yeasts can inhibit the growth of molds.

Enteric bacteria isolated in this study were *Klebsiella oxytoca* and *Enterobacter amnigenus*. It could be seen that as pH decreased and total titratable acidity increased, the presence of enteric bacteria decreased as they are acid intolerant, and at the 48 h of steeping, no enteric bacteria was isolated from all the fermentation setups. This was also corroborated by reports of Oluwafemi and Adetunji (2011) and Omemu and Faniran (2011).

The *Bacillus* species isolated in this study include *Bacillus formis* and *Bacillus subtilis*. Previous reports (Nwogwugwu et al., 2012; Nwokoro & Chukwu, 2012; Ogbulie, 1991) have also isolated *Bacillus* species in the fermentation of maize for ogi production, and they have been reported to show saccharolytic activities (Nwokoro & Chukwu, 2012). These organisms persisted toward the end of the fermentation indicating that they continued the saccharification of maize starch to release sugars. Corynebacteria (*C. kutscheri, C. striatum, and C. afermentans*) were observed at the beginning of fermentation, and according to Moslehi‐Jenabian, Lindegaard, and Jespersen (2010), they were responsible for the diastolic action necessary for the growth of lactic acid bacteria and yeasts.

## 5 CONCLUSION

In conclusion, the total heterotrophic plate count (THPC) in fortified maize: pigeon pea products was very high. This could constitute health hazards to infants as weaning foods. However, the microbial loads could be reduced through heat treatment as ogi is usually boiled or treated with boiled water before consumption and it can therefore be concluded that the fortified maize: pigeon pea products could be used as weaning foods.
